# Spatial resolution and peak-pressure-change measurement accuracy

**DOI:** 10.1186/1757-1146-5-S1-O30

**Published:** 2012-04-10

**Authors:** Todd C Pataky

**Affiliations:** 1 Department of Bioengineering, Shinshu University, Ueda, Nagano, 386-8567, Japan

## Background

It has been suggested that plantar pressures should be measured at ~6.2 mm to accurately characterize local maxima [1], and it has been shown that sensor widths of 10 mm can cause a 30% pressure underestimation at the metatarsal heads [2]. However, these results assume that pressure maxima, not maxima changes, are of primary interest. The purpose of this study was to examine how spatial resolution affects accuracy when measuring local maxima vs. changes in local maxima.

## Methods and materials

**Figure 1:**
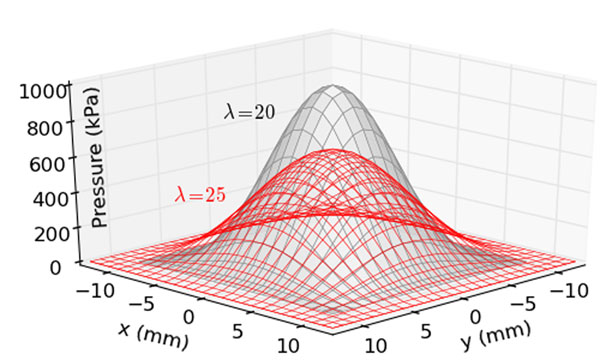
Pressure model (Eqn.1). Example pulses of λ=20 and 25 mm, with maxima of p*=1000 kPa and 640 kPa, Total force = 100 N.

A pressure pulse model (Figure 1) was generalized from [2] for force (*F*) and wavelength (*λ*) as:(1)

where the maximum pressure (p*) is 4*Fλ*^-2^, and the measured pressure (*p*) has an analytical solution dependent on sensor width *w*. The measurement accuracy of local-maxima and local-maxima-changes are *p*/p* [2], and (*p*_1_-*p*_2_)/(p*_1_-p*_2_), respectively, where ‘1’ and ‘2’ denote pulses with different wavelengths. To mimic insole-padding intervention, where total force is not expected to change, *F* was a constant 100 N and *λ* was varied from 20 mm [2]. Numerical optimization was used to find the critical sensor width that yielded various target accuracies for both local-maxima and a variety of local-maxima-changes (-100% to +100% change).

## Results

**Figure 2:**
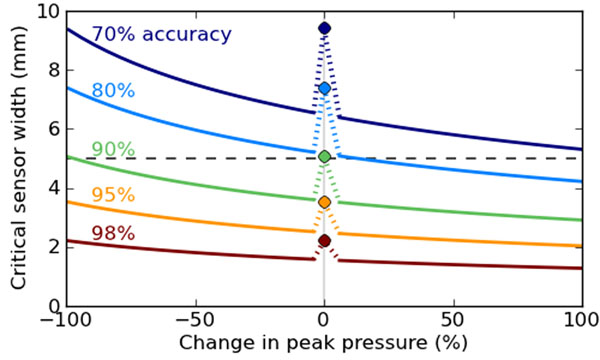
Critical sensor width needed to achieve given accuracies for local maxima (solid dots), and maxima changes (solid lines).

Results reveal that a target accuracy of 90% requires 5 mm resolution for local peak pressures (Figure 2), and that pressure-changes at 90% accuracy require resolutions of 4.1 mm and 3.2 mm, for changes of -50% and +50%, respectively. The reason is intuitive: the true difference pulse has higher frequency components than the original pulses, so pressure-change accuracy will be lower for all changes >-100%.

## Conclusion

This study has shown that, to achieve a given measurement accuracy, higher spatial resolutions are needed to measure local-pressure-maxima-changes than single-maxima. The main limitations are that pressure pulses are not, in general, constrained to have constant force and that broader (i.e. non-local) pressure changes were not considered.
